# Synthesis of silver nanoparticles with high efficiency and stability by culture supernatant of Bacillus ROM6 isolated from Zarshouran gold mine and evaluating its antibacterial effects

**DOI:** 10.1186/s12866-022-02490-5

**Published:** 2022-04-11

**Authors:** Rostampour Esmail, Alihosseini Afshar, Milani Morteza, Akbarzadeh Abolfazl, Ebrahim Akhondi

**Affiliations:** 1 Department of Chemical Engineering, Central Tehran Branch, Islamic Azad University, Tehran, Iran; 2 Department of Medical Nanotechnology, Faculty of Advanced Medical Sciences, Tabriz University of Medical Sciences, Tabriz, Iran; 3 Infectious and Tropical Diseases Research Center, Tabriz University of Medical Sciences, Tabriz, Iran

**Keywords:** Antibacterial agent, *Bacillus*, Zarshouran gold mine, Silver nanoparticles, ROM6

## Abstract

**Background:**

The use of bacteria to synthesize nanoparticles as an environment-friendly method has recently been considered by researchers. Bacteria residing in different mines have shown high potential in the synthesis of metal nanoparticles due to their compatibility with the environment. The aim of this study was to evaluate the ability of Zarshouran gold mine bacteria to synthesize silver nanoparticles and their antibacterial activity.

**Methods:**

After isolation of mine bacteria and several screening steps, silver ion tolerant bacteria that were able to synthesize extracellular silver nanoparticles were isolated and the most suitable isolate was selected and sequenced. The characteristics, stability, and production efficiency of silver nanoparticles were evaluated using UV–vis spectrophotometry, DLS, TEM, FTIR, and X-ray diffraction analysis. Finally, the antibacterial effect of silver nanoparticles against pathogenic bacteria was investigated.

**Results:**

Among the eight silver-tolerant bacteria, isolate No. 6 had high antibacterial activity and high potential in the synthesis and stabilization of silver nanoparticles. Therefore, this isolate was selected for the next experiments. The results of 16S rDNA sequencing showed that this isolate is related to *Bacillus pumilus*. We registered in the NCBI Bank called ROM6 with access number MW440543. The DLS and TEM analysis showed that silver nanoparticles produced by this isolate were most spherical with a size of less than 25 nm and were stable for at least 180 days. The efficiency at concentrations less than 0.9 g/l silver nitrate was over 90% and the minimum inhibition concentration of nanoparticles was determined against *S. aureus, E. coli, P. aeruginosa,* and *A. baumannii* ranging from 1.4 to 5.6 µg/ml.

**Conclusion:**

We found that the bacteria residing in the gold mine have a high capacity for the synthesis of spherical and high stable silver nanoparticles with a strong antibacterial effect.

**Supplementary Information:**

The online version contains supplementary material available at 10.1186/s12866-022-02490-5.

## Introduction

Nanoparticles are a set of zero-dimensional atoms in various forms divided into two main organic and inorganic groups. Metal nanoparticles, as inorganic nanoparticles, exhibit very different physical and chemical properties than conventional bulk metal. Silver nanoparticles are considered as one of the most widely used nanoparticles by researchers today due to their unique properties such as high conductivity, chemical resistance, antibacterial, antiviral, antifungal, anti-angiogenic, and anti-inflammatory properties in various fields of medicine. These nanoparticles are also applicable to various industries such as agriculture, animal husbandry, cosmetics, and hygiene [1]. Due to the alarming spread of resistance to classical antimicrobial agents, and the antibacterial properties of metal nanoparticles, the use of these nanoparticles, including silver nanoparticles, in innovative therapies to combat antibiotic-resistant pathogens seems necessary [2]. Silver nanoparticle synthesis is a remarkable scientific breakthrough in nanotechnology that can be accomplished in various ways. In chemical methods, nanoparticles are synthesized using toxic and flammable chemicals as regenerative and stabilizing agents, leading to environmental problems [3]. On the other hand, the use of high amounts of energy to maintain the pressure and temperature required for reaction steps is one of the disadvantages of physical methods. Given the disadvantages of physical and chemical methods, biosynthesis is an alternative that implies safe, eco-friendly and green methods. In addition, unlike physicochemical methods that involve a two-step procedure (for reduction and stabilization of NPs), in biological methods, one-step production is used to synthesize NPs due to both reducing and stabilizing agents in the biological extracts [3]. The use of extracts from macro-organisms such as plants, algae, or microorganisms such as fungi, bacteria, and yeast reduces the risk caused by toxic substances to humans and ecosystems [4]. In addition, bacteria are easy to work with, multiply quickly and cheaply, and growth conditions such as temperature, oxygen, and incubation time are easily controlled [5]. In the Nanobiotechnology field, microorganisms are used as small factories for the production of nanomaterials. The use of bacteria compared to other microorganisms has been considered in recent years because of their ability to produce nanoparticles of varying sizes, shapes, and morphologies [6]. Synthesis of nanoparticles using different strains of bacteria can be done intracellularly or extracellularly, however extracellular synthesis is simpler and more economical [7, 8]. The various bacteria have different tolerances to silver ions [9]. On the other hand, native bacteria of heavy metal mines have gained high tolerance to heavy metal ions, including silver, over the years due to their compatibility with the environment. These bacteria appear to be good options for the synthesis of silver nanoparticles [10]. The results of several studies in the last twenty years show that the native bacteria of different mines have different capabilities regarding the efficiency, speed, and stability of silver nanoparticles produced. Therefore, these bacteria can be considered as useful reserves to obtain valuable isolates. The aim of this study was to find a native bacterium capable of producing silver nanoparticles with maximum efficiency, high stability, and strong antibacterial activity from Zarshouran gold mine.

## Methods

### Soil sampling and bacterial isolation

Soil samples donated by " Zarshouran Gold Mining and Mining Industries Development Company" were collected from a depth of 5 cm below the soil surface and placed in sterile containers and transfer to the laboratory. In order to transfer soil bacteria into the solution, serial dilutions (10^–1^ &10^–2^) were prepared for each sample and 0.01 g of silver nitrate was added (Merck, Germany) to 100 ml of each dilution. So, the concentration of silver nitrate reached in all dilutions 0.1 g/L. Then 0.5 mL of each soil dilution were Spread on free-NaCl LB agar (Merck, Germany) plates and incubated at 35ºC for 48 h. In order to prepare pure culture, based on morphological characteristics, size, color, and shape of the colony, sub-cultured on LB agar plates [11].

### Determination of bacterial tolerance to silver ions

To investigate the tolerance of bacterial isolates to silver ions, 4 ml of free-NaCl LB broth culture medium containing different concentrations of silver nitrate (1 to 13 µg/ml) was inoculated with an equal amount of suspension equivalent to 0.5 McFarland bacterium, and incubated at 35ºC for 24 h. Then, a small amount of the contents of all tubes were sub-cultured on free-NaCl LB agar plates, separately, and their growth was evaluated after incubation. Finally, these isolates were inoculated in the culture medium of Trypticase soy broth containing 20% glycerol and stored at -20 °C for further experiments [12].

### Bacterial screening for synthesis silver nanoparticles

The bacterial isolates were cultured on 50 ml of NaCl-free LB broth culture medium and incubated at 35 °C in a shaker incubator at 120 rpm for 48 h. After incubation time, they were centrifuged in a 50 ml falcon tube at 10,000 rpm for 10 min. For complete biomass separation, the supernatant was passed through a 0.22-micron syringe filter. To synthesize the silver nanoparticles, 1 ml of each bacterial culture's supernatant was added to the sterile test tubes. Then, by adding silver nitrate solution, the concentration of silver nitrate in the tubes reached 0.56 µg/ml. The test tubes were incubated at a neutral pH at 30 °C for 48 h. The observed color change from clear to brown in the bacterial supernatant was the initial confirmation of silver nanoparticles' production by the relevant bacterial supernatant [13]. In order to confirm the production of nanoparticles, the absorption of electromagnetic waves of solutions inside the tubes was investigated using a UV–vis spectrophotometer (CECIL 7250) in the range of 400 to 600 nm. The presence of absorption peaks confirmed silver nanoparticles' production, while the control sample showed no absorption in this range [14, 15].

### Stability of silver nanoparticles in suspension

The stability of synthesized nanoparticles was evaluated in terms of the suspension appearance, cloth-like deposits, and UV–Vis spectrometry, every ten days.

### Antibacterial activity of synthesized silver nanoparticles

To investigate the antibacterial effect and MIC determination, the broth microdilution method was used on 96-well plates. First, 150 µl of broth nutrient medium was added to each well. To remove silver nitrate ions, enzymes, and other agents present in the bacterial culture supernatant, silver nanoparticles were washed. For this purpose, the supernatant containing silver nanoparticles was poured into 1.5 ml microtubes and washed using 1 ml of sterile phosphate buffer, and centrifuged at 15,000 rpm for 15 min (three times), then dried at 60° C for 12 h. Then the serial dilutions of nanoparticles were prepared in 8 consecutive wells ranging from180-1.4 µg/ml. For microbial assay, the bacterial strains including *S. aureus, E. coli, P. aeruginosa and A. baumannii* purchased from Iranian Research Organization for Science and Technology (IROST), Persian Type Culture Collection. After culturing the 0.5 McFarland standard suspension of these strains was prepared in normal saline sterile. Bacterial suspensions were added in a ratio of 1:100 in wells containing broth and silver nanoparticles and incubation overnight. Then, 0.1 ml of each well content was subculture on Nutrient agar plates, and their growth was investigated after 48 h incubations. In each strain, the lowest concentration of silver nanoparticles in which no growth was observed was reported as MIC. All experiments were performed with triplicate and statistical calculations were performed with SPSS software. One well containing culture medium and bacteria without nanoparticles and the other well containing culture medium and nanoparticles without bacteria were used as positive and negative controls, respectively.

### Identification of the bacterial isolate No.6 by 16S rRNA sequencing

To identify isolate number 6, genomic DNA of the bacterium was extracted by the phenol–chloroform method [16]. The PCR was used to amplify the 16S rDNA region of the bacterium by forward U8F primers (5'AGAGTTTGATCCTGGCTCAG-3 ') and revers U1390R (5'GACGGGCGGTGTGTACAA-3') [16, 17]. After PCR, the product was sequenced by Bioneer Korea. The sequence obtained using NCBI BLAST software was compared in genetic similarity with other bacterial species in the genomic bank.

### Characterization of synthesized silver nanoparticles

Color changes in the culture medium after the formation of silver nanoparticles was considered the primary characteristic of nanoparticle synthesis. UV–Vis analysis was performed on a spectrophotometer (CECIL 7250) in the range of 400—600 nm after dilution and use of LB broth with silver nitrate as blank. To accomplish the FTIR the NPs suspensions were centrifuged at 10,000 rpm, the pellets of NPs were dried and powdered. Then, the samples' IR spectrum was obtained using FT-IR spectrometer (Bruker—Tensor 27) in the range of 500 to 4000 cm-1 wavenumber. Also, AgNPs suspensions were prepared as a fine powder and it was sent to the laboratory of Tabriz Geological Survey and the pattern of diffraction or diffractogram of the sample was obtained. The analysis of the obtained diffractogram compared to the reference JCPDS File No.: 01–1167 [17]**.** After sonication of silver nanoparticles () James Europe 6 MX, 10 min), their size and characterization were determined by DLS. Also, exact size and morphology were obtained using an electron microscope (TEM Philips EM 208S).

### The efficiency of the AgNPs bacterial biosynthesis by the No. 6 isolate

In order to determine the production efficiency of silver nanoparticles, all variables were kept constant, and in the same conditions, only the silver nitrate concentration was changed. For this purpose, 6 Erlenmeyer flakes containing 100 ml of solution with 33% by volume of bacterial supernatant (including 33 ml of bacterial supernatant and the rest of distilled water) were selected, and the silver nitrate concentration was changed from 0.5 g/L to 1 g/L. After 48 h, the mass of silver nanoparticles produced in each sample after washing and drying was measured, and was calculated based on the following formula.

$${\text{Efficiency = }}\frac{{{\text{Mass}}\;{\text{of}}\;{\text{AgNPs}}}}{{{\text{Mass}}\;{\text{of}}\;{\text{Silver}}}}*100$$

## Results

### Bacterial screening of potential AgNPs biosynthesizes

Pure cultures of eight bacterial isolates were obtained from soil samples and were tested for silver ions tolerance in free-NaCl LB culture medium containing concentrations of 1 to 13 g/L silver nitrate. The bacterial isolates that did not grow in LB agar were excluded. Thus, 5 isolates were used in subsequent experiments. As shown in Table 1, two isolates (6 and 7) had a high tolerance to silver nitrate.

**Table 1:** Comparison of isolate tolerance to different concentrations of silver ions

Bacterial isolates number	The concentration of silver nitrate g/l									
	1	5	6	7	8	9	10	11	12	13
1	-	-	-	-	-	-	-	-	-	-
2	-	-	-	-	-	-	-	-	-	-
3	+	-	-	-	-	-	-	-	-	-
4	+	-	-	-	-	-	-	-	-	-
5	+	+	+	-	-	-	-	-	-	-
6	+	+	+	+	+	+	-	-	-	-
7	+	+	+	+	+	+	+	+	+	+
8	-	-	-	-	-	-	-	-	-	-

- no growth, + growth

**Fig. 1:**
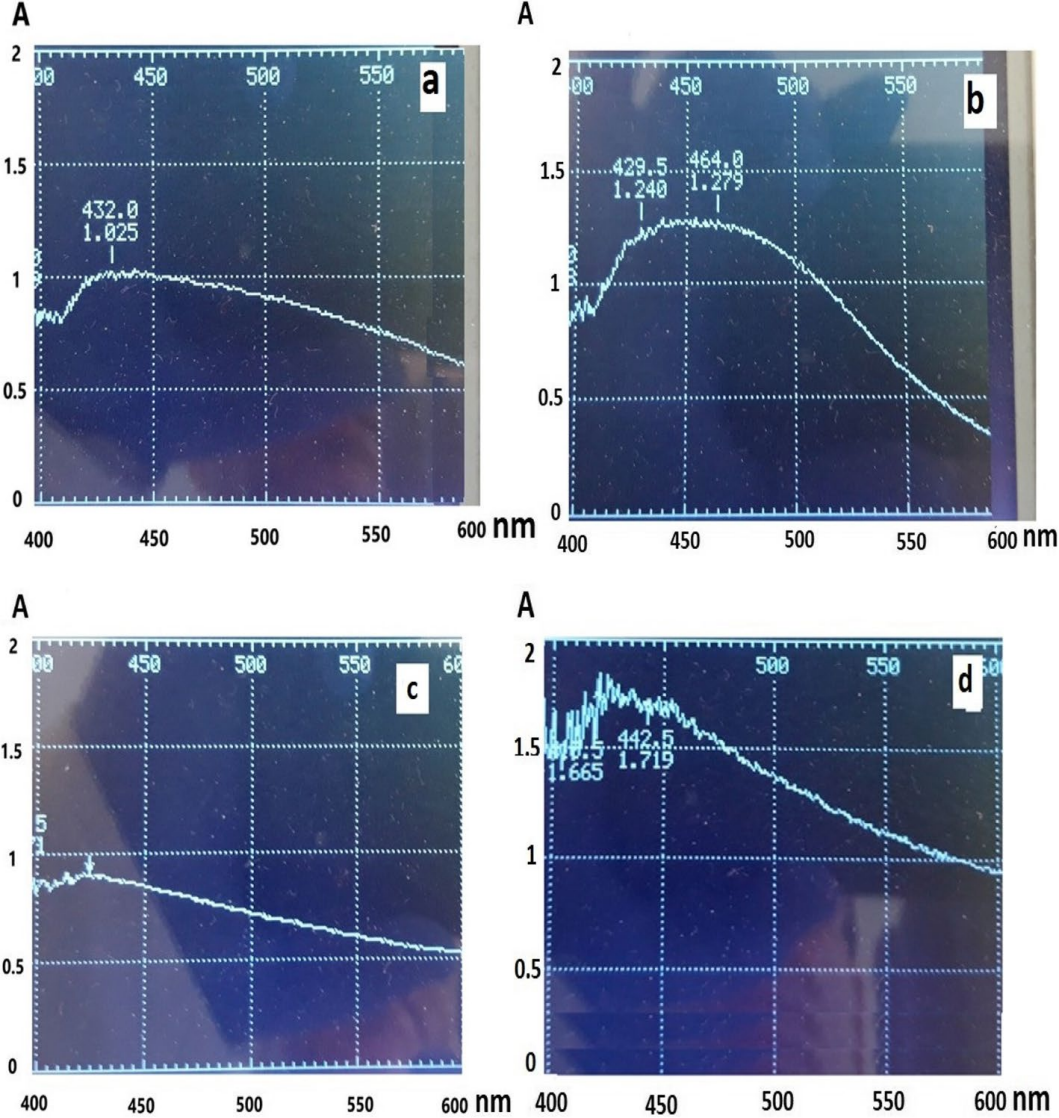
Absorption spectrum results of the AgNPs suspension related by isolates (**a**) No. 5, (**b**) No. 3, (**c**) No. 7, (**d**) No. 6

On four strains the color of the medium changed to brown, which was the initial confirmation of silver nanoparticles' production by the supernatant of these bacteria. We found the color intensity was different in distinct isolates (supplementary 1). The absorption spectrum results of the AgNPs suspension in the range of 400 to 600 nm, are shown in Fig. 1. The absorption peaks in the range of 400–470 nm showed the synthesis of silver nanoparticles in isolates No. 3, 5, 6, and 7 which confirmed the macroscopic color change. Similarly, isolates No. 6 and 7 had the highest and the lowest absorption, respectively. Also, isolate No. 4 was unable to synthesize silver nanoparticles, so it was excluded.

The MIC results of silver nanoparticles synthesized by four bacterial cultures against pathogenic bacterial strains were determined from 1.4 to 45 μg/ml (Table 2). The inhibitory potential of nanoparticles synthesized by the No. 6 isolate was better than other isolates (MIC = 1.4–5.6 µg/ml). The greatest antibacterial effect of synthesized nanoparticles was observed on *E. coli* and *A. baumannii****.***

**Table 2:** The MIC of synthesised nanoparticles against pathogenic strains

	MIC of Synthesised nanoparticles (µg/ml)			
Pathogenic strains	isolate No. 3	isolate No.5	isolate No.6	isolate No.7
*Escherichia coli*	11.25	1.4	1.4	45
*Pseudomonas aeruginosa*	22.5	22.5	2.8	45
*Acinetobacter baumannii*	5.6	5.6	1.4	11.25
*Staphylococcus aureus*	11.25	2.8	5.6	22.5

**Fig. 2:**
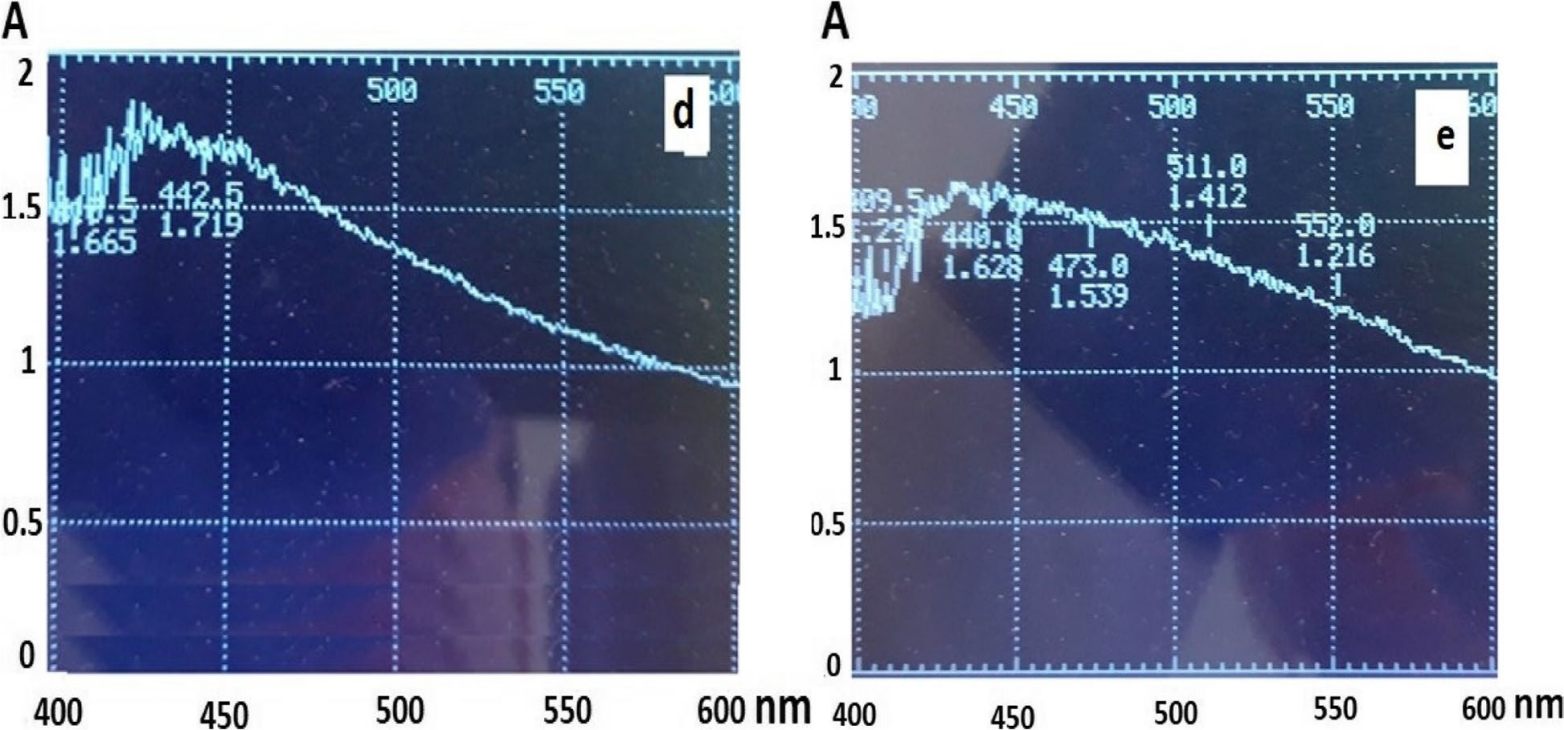
UV–Vis analysis of AgNPs suspension by isolate No. 6 (d) Initial (e) 180 days later

We found the stability of nanoparticles synthesized by NO.6 isolate was higher than other isolates and at least 180 days under light and laboratory temperature conditions (Table 3 and Fig. 2). This was indicated the high potential of the isolate to synthesize silver nanoparticles.

**Table 3:** Comparison of stability silver nanoparticles

isolate No	3	5	6	7
Daily stability of synthesized nanoparticles (day)	50	30	180	10

The study of silver ion tolerance, the ability to synthesize and stabilize silver nanoparticles, and its antibacterial activity (Tables 1, 2, 3) showed that No. 6 was the most suitable isolate for the next experiments.

### Identification of isolate No. 6 by 16 s rDNA sequence analysis

The isolate No. 6 was sequenced by the National Centre for Genetic and Biological Reserves of Iran and Bioneer Korea Company, as described in supplementary 2. It was found that this isolate had 99.9% similarity with sequences of *Bacillus Pumilus* ATCC 7061 (T) with access number ABRX01000007. We registered this isolate in the NCBI Bank called ROM6 with access number MW440543.

### Characterization of nanoparticles

**Fig. 3:**
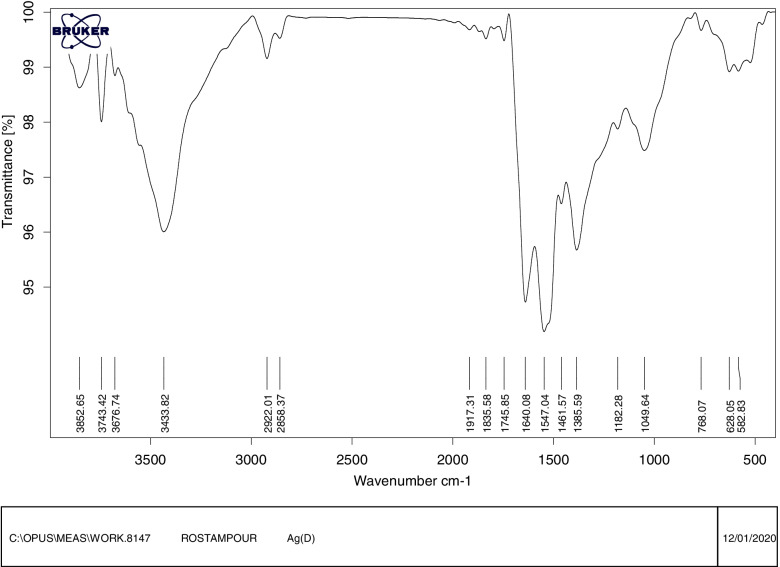
FTIR spectrum nanoparticles- isolate NO.6

The spectrum of FTIR nanoparticles in the wavelength range of 500 to 4000 cm-1 was studied. The recorded FTIR spectrum for silver nanoparticles indicates that the peaks at 3400–3700 cm^−1^ may be due to the presence of the –NH or –OH group, and the observed bands show from 2800 to 3000 cm-1 give the vibration traction of C- H functional groups. Also, the observed bands from 1700 to 2000 cm-1 are probably due to the C = O functional groups' vibration traction. The peak at 1640 cm-1 show the presence of CN and carbonyl in the proteins, and the observed bands from 1500 to 1000 cm-1 probably indicate the tensile vibrations of the C-O and C = C functional groups (Fig. 3) [18].

**Fig. 4:**
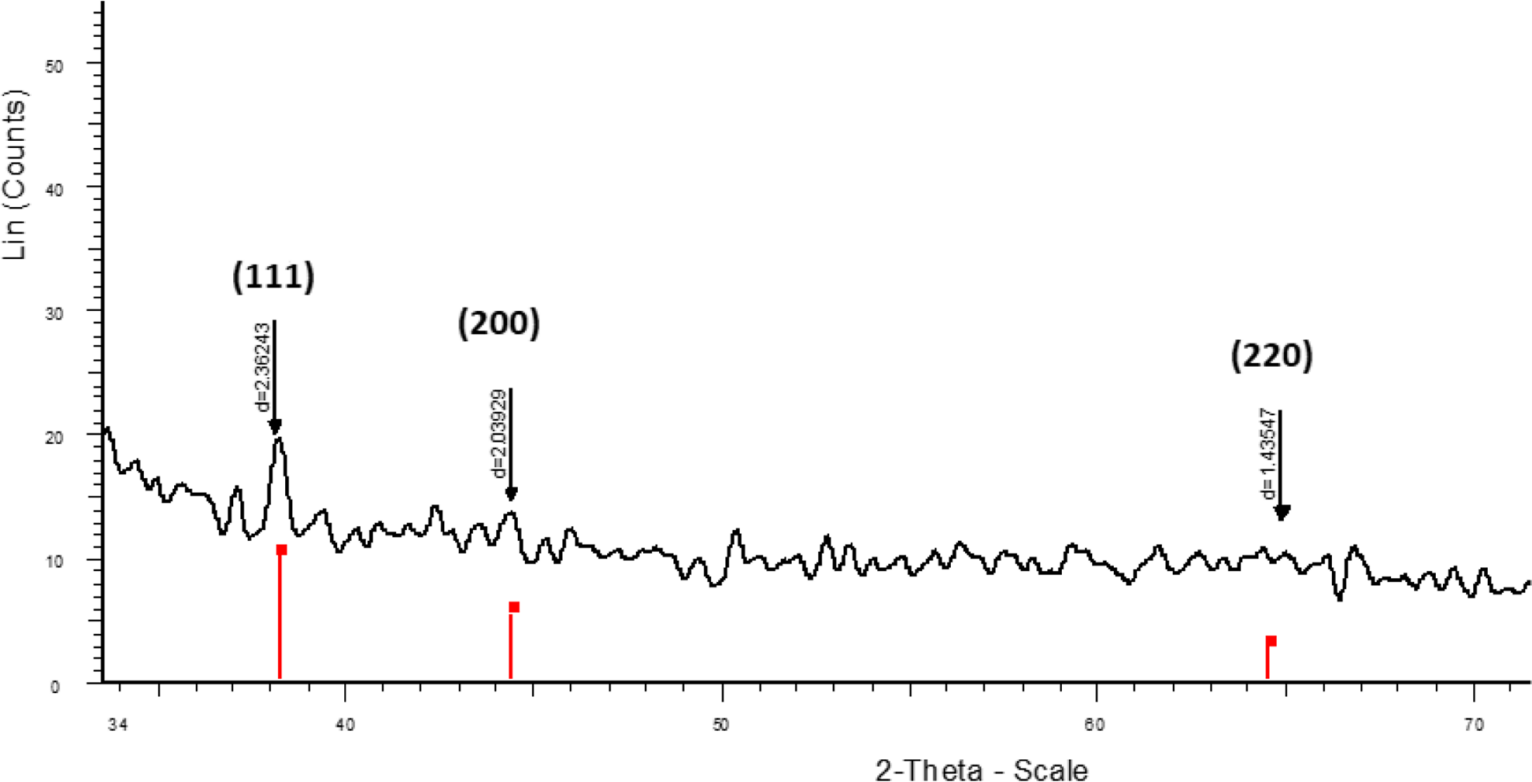
The X-ray diffraction of AgNPs biosynthesized by the bacterial isolate No. 6

The XRD spectrum of nanoparticles was recorded to characterize nanoparticles and confirm the particles as silver. As illustrated in Fig. 4, three distinct diffraction peaks at 2θ = 38.12°, 44.23° and 64.32 appeared, which index the plans (111), (200) and (220) of silver and are label in Fig. 3. This is closely similar to the announced reference JCPDS File No: 01–1167 [17].

**Fig. 5:**
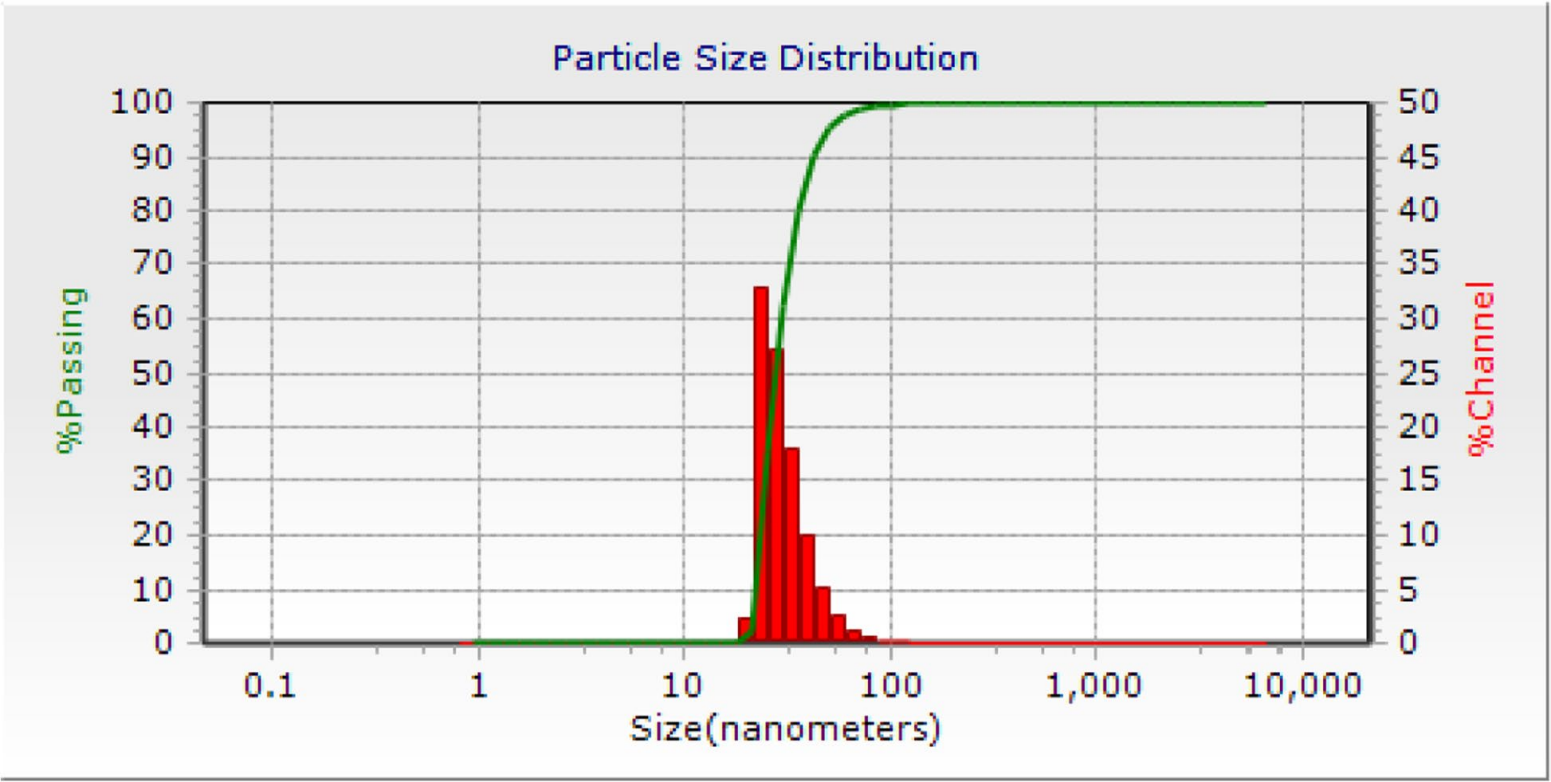
The PSD of the synthesized nanoparticles- isolate NO.6

Particle size were determined using DLS spectroscopy or dynamic light scattering technique (Fig. 5). Studying the PSD of the synthesized nanoparticles, it was determined that the average particle size distribution was between 20 and 70 nm, and the highest particle size percentage was around 27.87 nm. In addition, the synthesized nanoparticles were almost mono disperse.

**Fig. 6:**
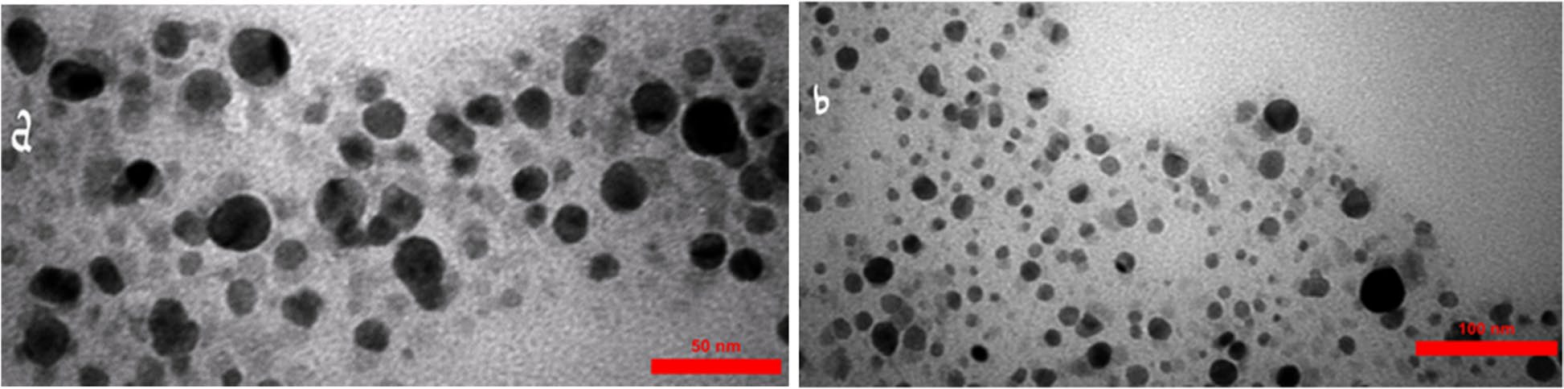
The TEM analysis of the synthesized nanoparticles by isolate NO. 6

The TEM result showed actual size of synthesized nanoparticles was less than 25 nm and spherical shape (Fig. 6).

In this study, the conversion efficiency of ions to nanoparticles for a given amount of supernatant was calculated as an important factor. Therefore, we reported that 33 ml of the supernatant could convert a few percent of silver ions into nanoparticles. The results showed that firstly, with increasing the concentration of silver ions, the synthesis efficiency decreased. Secondly, with 33% by volume of supernatant at concentrations less than 0.9 g/l silver nitrate, the synthesis efficiency was more than 90% (Table 4).

**Table 4:** Synthesis efficiency of nanoparticles with 33% by volume of supernatant

Silver nitrate mass per 100 ml of solution (g)	0.05	0.06	0.07	0.08	0.09	0.1
Silver nitrate concentration (g/l)	0.5	0.6	0.7	0.8	0.9	1
Silver ion (g)	0.032	0.038	0.044	0.050	0.057	0.063
Nano Silver (g)	0.031	0.037	0.042	0.046	0.052	0.054
Nanoparticle synthesis efficiency (%)	97	97	95	92	91	86

## Discussion

Nowadays, silver nanoparticles have been considered as one of the most widely used nanoparticles by researchers. Old nanoparticle production methods, such as physical and chemical methods, need to be revised due to fundamental problems such as environmental and energy issues [19–21]. An alternative method that has been studied by researchers in the last twenty years is the bio-production method. In the bioproduction method, plants or microorganisms are used to produce nanoparticles. There are several reasons for the use of bacteria compared to other microorganisms in recent years, including ease to use, growth rapid, and cheaper proliferation. In bacteria, growth conditions such as temperature, oxygen, and incubation time can be easily controlled. Also, due to the lack of a specific nucleus, genetic manipulation is easy in them, which leads to the achievement of industrial strains with higher production efficiency [4, 6]. Native bacteria of heavy metal mines have adapted to the environment contaminated with these heavy metals over the years and have become more tolerant of these metals [22]. Studies by several researchers have shown that these bacteria are valuable potential candidates for synthesizing heavy metal nanoparticles such as silver nanoparticles [1, 9, 12]. In this study, the Zarshouran gold mine isolates, that were more tolerant to silver ions were selected for the extracellular synthesis of nanoparticles using supernatant. The highly tolerable isolates have been used in several similar studies to synthesize silver nanoparticles [1, 9, 12]. The solution's color change after silver nanoparticles' production may be due to vibrations of the plasmon surface [23]. Our knowledge of the exact mechanism of the extracellular synthesis of silver nanoparticles is not yet complete. It seems that proteins and enzymes such as nitrate reductase in supernatant play important roles in reducing silver ion [24, 25]. To compare the antibacterial activity of nanoparticles, MIC was determined in four bacterial human pathogens. Our results revealed low MIC values (about 1.4 µg/mL) for two important pathogens ( *E. coli* and *A. baumannii*); this value is lower compared with reported values in similar studies, which reports MIC values between 6.25 to 50 µg/ml [9, 26]. These results are according to previous studies, which proposed that silver nanoparticles produced by microorganisms have a wide range of antimicrobial effects on gram-positive and gram-negative bacteria [13, 27, 28]. The antimicrobial activity mechanism of silver nanoparticles is not fully understood. But they may attach to the surface of the cell membrane and disrupt its physiological functions by creating pores or possibly causing reactive oxygen species (ROS), impaired respiratory function, and interfering with DNA replication [29, 30]. However, other researchers have suggested several mechanisms, including inactivation of the cell proteins, disruption of genetic material, and enzymes degradation [31]. Silver may have a greater affinity for reacting with biomolecules containing sulfur and phosphorus in the cell. Thus, sulfur-containing proteins in cell membranes or within cells, as well as phosphorus-containing elements such as DNA, are the site of choice for the binding of silver nanoparticles [32]. The 180-day stability of silver nanoparticles synthesized with isolate No. 6 shows this isolate high potential for silver nanoparticles production and the various possible applications of this nanoparticle. Our synthesized nanoparticles showed high stability compared to the results of similar studies [9, 11, 17]. The presence of proteins secreted in bacterial supernatants may stabilize the synthesized nanoparticles. These proteins can bind to nanoparticles through free amine groups or residual cysteine and prevent the accumulation [33, 34]. The FTIR spectrum showed that the amine and amide bonds and various functional groups such as carboxylic and hydroxyl were attached with silver and possibly stabilizing the nanoparticles and preventing their accumulation by proteins or polysaccharides [18]. The X-ray diffraction analysis showed that the synthesized nanoparticles were made of metallic silver. Many peaks were observed in the spectrum, which may be due to crystalline biological compounds on the nanoparticles surface [17]. The DLS and TEM study of the synthesized nanoparticles showed the synthesized nanoparticles exact size and morphology. DLS measures the size distribution and particle size characteristics by measuring random changes in light intensity scattered from a suspension or solution [35]. TEM analysis confirmed the DLS results. It also showed that our synthesized nanoparticles' actual size was less than 25 nm and that they were most spherical and almost uniform. Previous studies have shown that silver nanoparticles produced by various bacteria with different sizes and shapes. Such as synthesis of 93 nm cuboidal nanoparticles by *S. maltophilia*, synthesis of 10–40 nm quasispherical nanoparticles by *Morganella spp*. and synthesis of 14.86 nm triangular, rod-shaped, spherical nanoparticles by *X. oryzae* [36–38]. Also, silver nanoparticles produced by various strains of Bacillus have a spherical shape, and their size is from 4 to 94 nm. Considering that isolate No. 6 studied was a Bacillus species, the results obtained are consistent with the results of other researchers [9, 13, 30]. Calculation of conversion efficiency is one of the important factors for the mass production of silver nanoparticles using bacterial supernatant. We have not seen such a calculation in any study. The ability of this isolate to convert more than 90% of silver ions into nanoparticles at concentrations less than 0.9 g/L of silver nitrate raises great hopes for mass and industrial production of silver nanoparticles using the supernatant of this isolate.

## Conclusion

In this study, a new species of *Bacillus* was introduced, which was isolated from Zarshouran gold mine. Also, the high potential of this isolate in the biosynthesis of silver nanoparticles was investigated. This isolate produces nanoparticles in concentrations less than 0.9 g/L silver nitrates with an efficiency of over 90%. Nanoparticles produced with this isolate are most spherical and almost monodisperse with sizes less than 25 nm. These nanoparticles not only have high stability and are stable for at least 180 days but also have high antimicrobial activity. Therefore, this isolate can be considered as a suitable candidate for the industrial production of silver nanoparticles for application to different areas of nanotechnology, especially medical applications.

## Supplementary Information

**Additional file 1.** Supplementary file 1.

**Additional file 2.** Supplementary file 2.

## Data Availability

The datasets used and/or analyzed during the current study are available from the corresponding author on reasonable request. The Bacterial sequence data introduced in this study (ROM6) with access number MW440543 is available in the below address. (https://www.ncbi.nlm.nih.gov/nuccore/MW440543.1)

## Abbreviations

AgNPs: Silver nanoparticles
DLS: Dynamic Light Scattering
TEM: Transmission Electron Microscope
FTIR: Fourier Transform Infrared Spectrometer
LB: Luria -Bertani culture medium
MIC: Minimum inhibitory concentration
PSD: Particle Size Distribution
JCPDS: Joint Committee on Powder Diffraction Standards
BLASTX: https://blast.ncbi.nlm.nih.gov/Blast.cgi?PROGRAM

## References

[1] Karkaj OS, Salouti M, Sorouri R, Derakhshan FK (2012). Biosynthesis of silver nanoparticles by Bacillus bacteria Biosynthesis of silver nanoparticles by Bacillus bacteria. Pajoohandeh J.

[2] Saravanan M, Jacob V, Arockiaraj J, Prakash P (2014). Extracellular biosynthesis, characterization and antibacterial activity of silver nanoparticles synthesized by Bacillus subtilis (NCIM—2266). J Bionanoscience.

[3] Divya K, Kurian LC, Vijayan S, Manakulam Shaikmoideen J (2016). Green synthesis of silver nanoparticles by Escherichia coli: Analysis of antibacterial activity. J Water Environ Nanotechnol.

[4] Pantidos N, Horsfall LE (2014). Biological synthesis of metallic nanoparticles by bacteria, fungi and plants. J Nanomedicine Nanotechnol.

[5] Barabadi H, Honary S, Ebrahimi P, Alizadeh A, Naghibi F, Saravanan M (2019). Optimization of myco-synthesized silver nanoparticles by response surface methodology employing Box-Behnken design. Inorganic Nano-Metal Chem.

[6] Zhang X, Yan S, Tyagi R, Surampalli R (2011). Synthesis of nanoparticles by microorganisms and their application in enhancing microbiological reaction rates. Chemosphere.

[7] Shahverdi AR, Fakhimi A, Shahverdi HR, Minaian S (2007). Synthesis and effect of silver nanoparticles on the antibacterial activity of different antibiotics against Staphylococcus aureus and Escherichia coli. Nanomedicine: Nanotechnol Biol Med.

[8] Sriram MI, Kanth SB, Kalishwaralal K, Gurunathan S (2010). Antitumor activity of silver nanoparticles in Dalton’s lymphoma ascites tumor model. Int J Nanomedicine.

[9] Khaleghi M, Khorrami S, Ravan H (2019). Identification of Bacillus thuringiensis bacterial strain isolated from the mine soil as a robust agent in the biosynthesis of silver nanoparticles with strong antibacterial and anti-biofilm activities. Biocatalysis Agric Biotechnol.

[10] Gumel A, Surayya M, Yaro M, Waziri I, Amina A (2019). Biogenic synthesis of silver nanoparticles and its synergistic antimicrobial potency: an overview. J Appl Biotechnol Bioeng.

[11] Ashengroph M (2014). Extracellular Synthesis of Silver Nanoparticles by Ralstonia sp. SM8 Isolated from the Sarcheshmeh Copper Mine. Biol J Microorganism.

[12] Ashengroph M. Extracellular Synthesis of Silver Nanoparticles by Ralstonia sp. SM8 Isolated from the Sarcheshmeh Copper Mine. Biol J Microorganism. 2014;3(9):53-64.

[13] Pourali P, Yahyaei B (2016). Biological production of silver nanoparticles by soil isolated bacteria and preliminary study of their cytotoxicity and cutaneous wound healing efficiency in rat. J Trace Elem Med Biol.

[14] Krishnaraj C, Jagan E, Rajasekar S, Selvakumar P, Kalaichelvan P, Mohan N (2010). Synthesis of silver nanoparticles using Acalypha indica leaf extracts and its antibacterial activity against water borne pathogens. Colloids Surf B Biointerfaces.

[15] Hoseynzadeh A, Khaleghi M, Sasan H (2017). Investigating the Antimicrobial Effects of Silver Nanoparticles Synthesized by Bacteria Isolated From Agricultural Soils of Kerman, Iran. Iranian J Med Microbiol.

[16] Milani M, Ghotaslou R, Somi MH, Rafeey M, Akhi MT, Nahaei MR (2012). The status of antimicrobial resistance of Helicobacter pylori in Eastern Azerbaijan, Iran: comparative study according to demographics. J Infect Chemother.

[17] Anjum S, Abbasi BH (2016). Thidiazuron-enhanced biosynthesis and antimicrobial efficacy of silver nanoparticles via improving phytochemical reducing potential in callus culture of Linum usitatissimum L. Int J Nanomedicine.

[18] Deka AC, Sinha SK (2015). Mycogenic silver nanoparticle biosynthesis and its pesticide degradation potentials. Int J Technol Enhancements Emerg Eng Res.

[19] Dahoumane SA, Jeffryes C, Mechouet M, Agathos SN (2017). Biosynthesis of inorganic nanoparticles: A fresh look at the control of shape, size and composition. Bioeng.

[20] Dahoumane SA, Mechouet M, Wijesekera K, Filipe CD, Sicard C, Bazylinski DA (2017). Algae-mediated biosynthesis of inorganic nanomaterials as a promising route in nanobiotechnology–a review. Green Chem.

[21] Anastas PT, Kirchhoff MM (2002). Origins, current status, and future challenges of green chemistry. Acc Chemical Res.

[22] Silver S (2003). Bacterial silver resistance: molecular biology and uses and misuses of silver compounds. FEMS Microbiol Rev.

[23] Patil MP, Kim G-D (2017). Eco-friendly approach for nanoparticles synthesis and mechanism behind antibacterial activity of silver and anticancer activity of gold nanoparticles. Appl Microbiol Biotechnol.

[24] Wang L, Zhang H, Rehman MU, Mehmood K, Jiang X, Iqbal M (2018). Antibacterial activity of Lactobacillus plantarum isolated from Tibetan yaks. Microb Pathog.

[25] Narayanan KB, Sakthivel N (2010). Biological synthesis of metal nanoparticles by microbes. Adv Colloid Interface Sci.

[26] Al-Bahrani R, Raman J, Lakshmanan H, Hassan AA, Sabaratnam V (2017). Green synthesis of silver nanoparticles using tree oyster mushroom Pleurotus ostreatus and its inhibitory activity against pathogenic bacteria. Mater Lett.

[27] Barros CH, Fulaz S, Stanisic D, Tasic L (2018). Biogenic nanosilver against multidrug-resistant bacteria (MDRB). Antibiot.

[28] Nanda A, Saravanan M (2009). Biosynthesis of silver nanoparticles from Staphylococcus aureus and its antimicrobial activity against MRSA and MRSE. Nanomedicine: Nanotechnol, Biol Med..

[29] Guzmán MG, Dille J, Godet S (2009). Synthesis of silver nanoparticles by chemical reduction method and their antibacterial activity. Int J Chem Biomol Eng.

[30] Shanthi S, Jayaseelan BD, Velusamy P, Vijayakumar S, Chih CT, Vaseeharan B (2016). Biosynthesis of silver nanoparticles using a probiotic Bacillus licheniformis Dahb1 and their antibiofilm activity and toxicity effects in Ceriodaphnia cornuta. Microb Pathog.

[31] Patra JK, Baek K-H (2017). Antibacterial activity and synergistic antibacterial potential of biosynthesized silver nanoparticles against foodborne pathogenic bacteria along with its anticandidal and antioxidant effects. Front Microbiol.

[32] Priyadarshini S, Gopinath V, Priyadharsshini NM, MubarakAli D, Velusamy P (2013). Synthesis of anisotropic silver nanoparticles using novel strain, Bacillus flexus and its biomedical application. Colloids Surf B Biointerfaces.

[33] Khorrami S, Zarrabi A, Khaleghi M, Danaei M, Mozafari M (2018). Selective cytotoxicity of green synthesized silver nanoparticles against the MCF-7 tumor cell line and their enhanced antioxidant and antimicrobial properties. Int J Nanomedicine.

[34] Nayak D, Ashe S, Rauta PR, Kumari M, Nayak B (2016). Bark extract mediated green synthesis of silver nanoparticles: evaluation of antimicrobial activity and antiproliferative response against osteosarcoma. Mater Sci Eng: C.

[35] Amini N, Amin G, Jafari Azar Z (2017). Green synthesis of silver nanoparticles using Avena sativa L. extract. Nanomedicine Res  J.

[36] Oves M, Khan MS, Zaidi A, Ahmed AS, Ahmed F, Ahmad E (2013). Antibacterial and cytotoxic efficacy of extracellular silver nanoparticles biofabricated from chromium reducing novel OS4 strain of Stenotrophomonas maltophilia. PloS one.

[37] Parikh RY, Singh S, Prasad B, Patole MS, Sastry M, Shouche YS (2008). Extracellular synthesis of crystalline silver nanoparticles and molecular evidence of silver resistance from Morganella sp.: towards understanding biochemical synthesis mechanism. ChemBioChem.

[38] Narayanan KB, Sakthivel N (2013). Biosynthesis of silver nanoparticles by phytopathogen Xanthomonas oryzae pv. oryzae strain BXO8. J Microbiol Biotechnol.

