# Supplementary figures and images for: Synthesis of silver nanoparticles with high efficiency and stability by culture supernatant of Bacillus ROM6 isolated from Zarshouran gold mine and evaluating its antibacterial effects
Source: BMC Microbiol. 2022 Apr 11;22:97. doi: 10.1186/s12866-022-02490-5 (PMC8996393; doi:10.1186/s12866-022-02490-5)

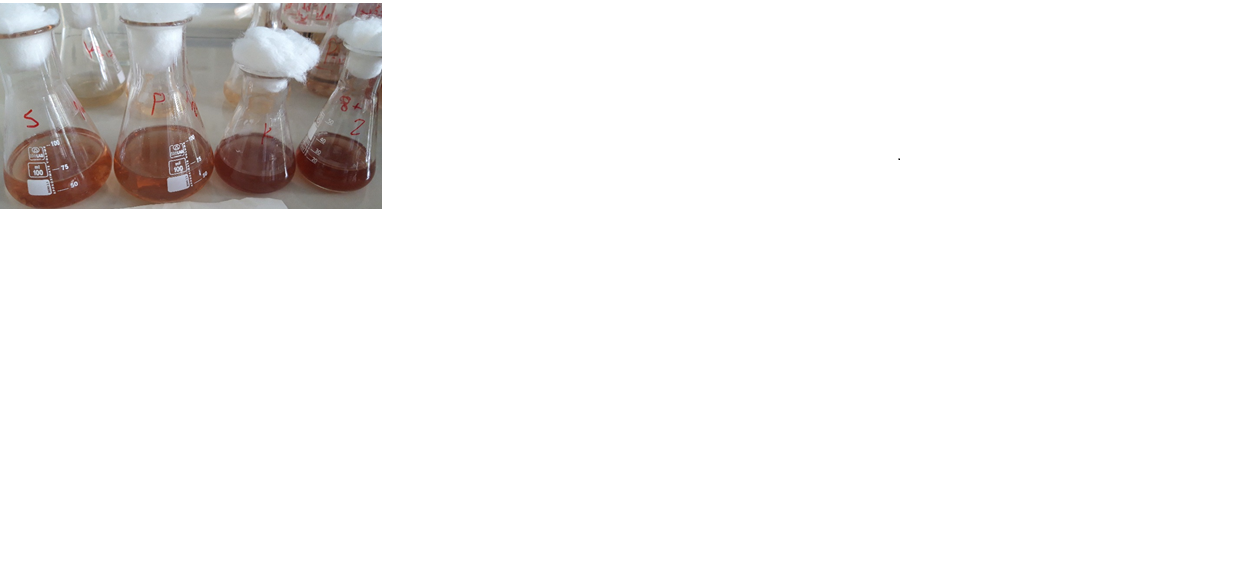

Supplement: Supplementary file 1 — Additional file 1. Supplementary file 1. [file 12866_2022_2490_MOESM1_ESM.png]
